# Hepatitis A Virus Infections Among Men Who Have Sex with Men — Eight U.S. States, 2017–2018

**DOI:** 10.15585/mmwr.mm7024a2

**Published:** 2021-06-18

**Authors:** Monique A. Foster, Megan G. Hofmeister, Justin P. Albertson, Kerri B. Brown, Alexis W. Burakoff, Ami P. Gandhi, Rosie E. Glenn-Finer, Prabhu Gounder, Po-Yi Ho, Tracy Kavanaugh, Julia Latash, Rebecca L. Lewis, Atkinson G. Longmire, Angela Myrick-West, Dana M. Perella, Vasudha Reddy, Emma S. Stanislawski, Juliet E. Stoltey, Susan M. Sullivan, Okey F. Utah, Jennifer Zipprich, Eyasu H. Teshale

**Affiliations:** ^1^Division of Viral Hepatitis, National Center for HIV/AIDS, Viral Hepatitis, STD, and TB Prevention, CDC; ^2^North Carolina Department of Health and Human Services;^ 3^Colorado Department of Public Health and Environment; ^4^Epidemic Intelligence Service, CDC^ 5^Georgia Department of Public Health; ^6^California Department of Public Health; ^7^Los Angeles County Department of Public Health, Los Angeles, California; ^8^New York City Department of Health and Mental Hygiene, New York; ^9^Virginia Department of Health; ^10^Philadelphia Department of Public Health, Pennsylvania; ^11^Maryland Department of Health; ^12^San Francisco Department of Public Health, California; ^13^Minnesota Department of Health.

During 1995–2011, the overall incidence of hepatitis A decreased by 95% in the United States from 12 cases per 100,000 population during 1995 to 0.4 cases per 100,000 population during 2011, and then plateaued during 2012─2015. The incidence increased by 294% during 2016–2018 compared with the incidence during 2013–2015, with most cases occurring among populations at high risk for hepatitis A infection, including persons who use illicit drugs (injection and noninjection), persons who experience homelessness, and men who have sex with men (MSM) (*1*–*3*). Previous outbreaks among persons who use illicit drugs and MSM led to recommendations issued in 1996 by the Advisory Committee on Immunization Practices (ACIP) for routine hepatitis A vaccination of persons in these populations (*4*). Despite these long-standing recommendations, vaccination coverage rates among MSM remain low (*5*). In 2017, the New York City Department of Health and Mental Hygiene contacted CDC after public health officials noted an increase in hepatitis A infections among MSM. Laboratory testing* of clinical specimens identified strains of the hepatitis A virus (HAV) that subsequently matched strains recovered from MSM in other states. During January 1, 2017–October 31, 2018, CDC received reports of 260 cases of hepatitis A among MSM from health departments in eight states, a substantial increase from the 16 cases reported from all 50 states during 2013–2015. Forty-eight percent (124 of 258) of MSM patients were hospitalized for a median of 3 days. No deaths were reported. In response to these cases, CDC supported state and local health departments with public health intervention efforts to decrease HAV transmission among MSM populations. These efforts included organizing multistate calls among health departments to share information, providing guidance on developing targeted outreach and managing supplies for vaccine campaigns, and conducting laboratory testing of clinical specimens. Targeted outreach for MSM to increase awareness about hepatitis A infection and improve access to vaccination services, such as providing convenient locations for vaccination, are needed to prevent outbreaks among MSM.

This analysis included confirmed cases of hepatitis A among MSM whose symptoms began during January 1, 2017–October 31, 2018. During this period, community outbreaks of hepatitis A among persons reporting drug use or homelessness or both were identified in California, Kentucky, Michigan, and Utah (*1*,*6*). These persons were not included in this analysis because they were found to be infected with a different strain of hepatitis A virus and therefore deemed a separate outbreak. Confirmed cases were defined as those in which a patient had an illness consistent with acute viral hepatitis and jaundice (or elevated serum alanine aminotransferase [ALT] levels [>200 IU/L]) and a positive immunoglobulin M (IgM) antibody to HAV, or positive nucleic acid amplification test result in the absence of a more likely diagnosis. Local and state health department personnel interviewed patients using standard questionnaires and reviewed medical records to supplement demographic, clinical, and risk factor information. Analysis was conducted using SAS (version 9.4; SAS Institute). Data collection was directly related to disease control and was deemed not to be human subject research. This activity was reviewed by CDC and was conducted consistent with applicable federal law and CDC policy.^†^

Available serum specimens from patients who received a positive test result for HAV IgM antibodies were submitted for additional testing at CDC or the Viral and Rickettsial Disease Laboratory at the California Department of Public Health. These specimens were tested for HAV RNA by polymerase chain reaction, and amplicons were sequenced to characterize a 315-base pair fragment of the VP1/P2B region, which defines the genotype of the virus.

During January 1, 2017–October 31, 2018, a total of 260 cases of hepatitis A among MSM were reported across the following eight states: California, Colorado, Georgia, Maryland, New York, North Carolina, Pennsylvania, and Virginia. During the analysis period, these states reported 1,229 cases of hepatitis A with “no” or “unknown” MSM status to the National Notifiable Diseases Surveillance System. Among these states, the highest number of cases among MSM occurred in New York (39%), specifically New York City (31%), and California (24%) (Table). Illness onset dates were available for 258 of 260 cases (Figure). The median age of MSM patients was 32 years (range = 19–75 years). Among patients for whom detailed clinical information was available, the most frequently reported signs and symptoms were fatigue or malaise (171 of 193 [89%]), dark urine (205 of 240 [85%]), and jaundice (205 of 254 [81%]) (Table). Median laboratory values for ALT, aspartate aminotransferase, and total bilirubin were 2,285 IU/L, 1,015 IU/L, and 6.8 mg/dL, respectively (Table). Among 212 patients with available viral hepatitis coinfection data, five of 212 (2%) had evidence of past or current hepatitis B virus infection, and two of 212 (1%) had evidence of past or current hepatitis C virus infection. Among 72 patients whose HIV infection status was known, coinfection with HIV was reported in 26 (36%) patients. Forty-eight percent (124 of 258) were hospitalized for a median of 3 days. No deaths were reported. Twenty-one percent (54 of 253) of patients reported international travel during the incubation period, 24% (59 of 244) of patients reported injection or noninjection drug use during the incubation period, and 8% (15 of 187) of patients reported receiving ≥1 dose of hepatitis A vaccine (Table).

**TABLE:** Characteristics and risk factors for hepatitis A, by reported cases (n = 260) among men who have sex with men — eight U.S. States,* January 1, 2017–October 31, 2018

Characteristic	No. (%)
Median age (range), yrs	32 (19–75)
**Reporting state**	
New York	101 (39)
California	63 (24)
Colorado	20 (8)
North Carolina	20 (8)
Pennsylvania	20 (8)
Maryland	14 (5)
Virginia	12 (5)
Georgia	10 (4)
**Risk exposures, no. (row %)**	
MSM	260/260 (100)
International travel during incubation period	54/253 (21)
Injection or noninjection drug use during incubation period	59/244 (24)
≥1 dose hepatitis A vaccine^†^	15/187 (8)
Hepatitis B infection^§^	5/212 (2)
Hepatitis C infection^§^	2/212 (1)
HIV infection^¶^	26/72 (36)
**Clinical symptoms and disease outcome, no. (row %)**	
Fatigue/Malaise	171/193 (89)
Dark urine	205/240 (85)
Jaundice	205/254 (81)
Anorexia	171/251 (68)
Nausea	175/257 (68)
Abdominal pain	162/256 (63)
Vomiting	124/257 (48)
Fever	120/254 (47)
Light or clay-colored stools	96/222 (43)
Diarrhea	73/243 (30)
Hospitalized	124/258 (48)
Duration of hospitalization, median(range), days**	3 (0–10)
Died	0/260 (—)
**Laboratory data, no. of patients with available data, median (range)**	
ALT (n = 251)	2,285 (181–7,575)
AST (n = 240)	1,015 (78–9,154)
Total bilirubin (n = 232)	6.8 (0.5–21.7)
**Hepatitis A virus genotype IA strain**	
Total no. of patients with genotype IA strains	126 (100)
U.S. MSM cluster 1	43 (34)
RIVM-HAV16–090	30 (24)
VRD_521_2016	20 (16)
U.S. MSM cluster 2	13 (10)
V16–25801	4 (3)
Other	16 (13)

**Abbreviation**: ALT = alanine aminotransferase; AST = aspartate aminotransferase; MSM = men who have sex with men.

* States that reported cases analyzed in this report were California, Colorado, Georgia, Maryland, New York, North Carolina, Pennsylvania, and Virginia.

^†^ California only considered documented hepatitis A vaccine doses as evidence of prior vaccination, whereas other states included hepatitis A vaccination self-reported by patients.

^§^ Colorado did not report hepatitis B and C infections.

^¶^ California, Colorado, and New York did not report HIV infections.

** Information on duration of hospitalization was available for 108 of 260 cases among MSM.

**FIGURE:**
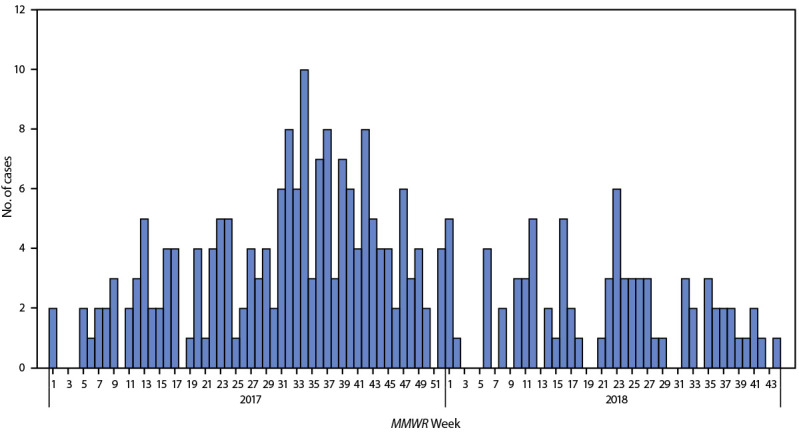
Hepatitis A virus infections (n = 258)* among men who have sex with men, by *MMWR* week† — eight U.S. states,**^§^** January 1, 2017–October 31, 2018 Abbreviation: MSM = men who have sex with men. * Dates of illness onset were available for 258 of 260 cases among MSM. ^†^ *MMWR* week numbering is sequential beginning with 1 and incrementing with each week to a maximum of 52 or 53 and is based on the epidemiologic week for disease reporting, which lasts Sundays through Saturdays. https://wwwn.cdc.gov/nndss/document/MMWR_week_overview.pdf ^§^ Cases were reported from the following eight states: California, Colorado, Georgia, Maryland, New York, North Carolina, Pennsylvania, and Virginia. Information obtained from case investigations did not allow for the definitive determination of where the outbreak started or how it progressed (e.g., via spread from one state to others or via simultaneous introduction of the involved hepatitis A virus strains in multiple states).

Anti-HAV IgM-positive specimens from 133 patients were submitted to the laboratories for HAV RNA isolation and additional strain characterization; all were positive for the presence of HAV RNA. Among these specimens, 95% (126 of 133) were HAV genotype IA, and 5% (six of 133) were HAV genotype IB; one specimen was determined to have an insufficient quantity for genotyping. Among the 126 specimens with HAV genotype IA sequences, 54 (43%) were from patients infected with a genotype IA strain that was genetically identical to one of three strains identified during recent HAV outbreaks among MSM in the European Union (30 [24%] RIVM-HAV16–090; 20 [16%] VRD_521_2016; and four [3%] V16–25801) (*7*). Two additional HAV genotype IA strains were circulating among MSM infected with HAV in the United States during the analysis period: MSM cluster 1 (43 [34%]) and MSM cluster 2 (13 [10%]) of the total 126 specimens (Table).

State and local health departments in affected areas provided outreach to the MSM community through websites and webinars, resources designed to reach the MSM community, targeted communication campaigns, and vaccination events in specialized venues.^§^ Letters and health alerts were sent to physicians to inform them of increases in HAV infections among MSM and remind them of the ACIP recommendation to vaccinate this population against hepatitis A. Many jurisdictions used social media outreach to target hepatitis A educational messaging and vaccination opportunities to MSM through such social platforms as Grindr, Scruff, and Facebook.^¶^^,^**

## Discussion

Hepatitis A outbreaks among MSM have been previously reported (*8*); evidence of increased risk of hepatitis A virus infection led the ACIP in 1996 to include MSM as a risk group that should receive hepatitis A vaccination (*4*). Despite this longstanding recommendation, vaccination coverage rates among MSM remain low. On the basis of 2013–2015 data from the National Health Interview Survey, the percentage of adult MSM in the noninstitutionalized U.S. population who reported ever having received a hepatitis A vaccination was 40% (*5*). Previous studies have determined that population immunity levels >70% are needed to prevent outbreaks among MSM (*9*).

HAV infections among MSM reported from eight states during January 1, 2017–October 31, 2018 contributed to the overall increase in hepatitis A incidence in the United States during 2016–2018 (*3*). During this period, coinfections with hepatitis B or hepatitis C viruses, hospitalization, and death were reported less frequently among MSM patients than among hepatitis A patients who reported drug use or homelessness (*1*). Among clinical specimens available for testing, 87% were infected with one of five HAV genotype IA strains: three of these five strains were observed in outbreaks associated with MSM that occurred during the same period (January 1, 2017–October 31, 2018) but in different parts of the world; to date the other two genotype IA strains were detected only in the United States (*7*).

Behaviors that facilitate HAV transmission among MSM vary and can involve sexual practices that enable fecal-oral transmission (e.g., digital-anal and oral-anal sex) (*7*). Case investigations of hepatitis A among MSM in the United States do not always reveal distinct sexual networks; anonymous involvement with sexual contacts makes partner notification and control of outbreak clusters difficult (*8*).

Hepatitis A vaccination is highly protective against HAV infection (*4*). Studies among African American MSM in the southern United States reported the strongest predictor for hepatitis A vaccination to be health care provider communication about patient sexual orientation and behaviors and low perceived barriers to vaccination. Perceived benefits of vaccination were not associated with increased vaccination (*10*).

The findings in this report are subject to at least four limitations. First, questions about sexual orientation and sexual practices on HAV case report forms are not standardized across jurisdictions. Second, the ability to draw conclusions from incomplete race and ethnicity data was limited, and therefore, not analyzed. Third, data regarding coinfections with other viruses, particularly HIV, were limited. Finally, distinguishing the cause of infection when persons reported multiple behaviors that increase risk of HAV infection was difficult and might have resulted in cases being misclassified 

Despite these limitations, this report highlights a gap in vaccination among MSM in the United States. Targeted outreach to MSM, including efforts that increase knowledge about hepatitis A infection and improve access to vaccination services, such as providing convenient locations for vaccination, are needed to improve hepatitis A immunity among MSM and to help prevent outbreaks.

**Summary**

**What is already known about this topic?**

Hepatitis A vaccination is recommended for men who have sex with men (MSM).

**What is added by this report?**

During January 1, 2017–October 31, 2018, a total of 260 cases of hepatitis A occurred among MSM from eight states compared with 16 cases reported from 50 states during 2013─2015. Forty-eight percent (124 of 258) of MSM patients were hospitalized for a median of 3 days. No deaths were reported.

**What are the implications for public health practice?**

Targeted outreach to increase awareness about hepatitis A infection and improve access to vaccination services are needed to prevent outbreaks among MSM.

## Footnotes

* CDC or the Viral and Rickettsial Disease Laboratory at the California Department of Public Health.

† 45 C.F.R. part 46.102(l)(2), 21 C.F.R. part 56; 42 U.S.C. Sect. 241(d); 5 U.S.C. Sect. 552a; 44 U.S.C. Sect. 3501 et seq.

§
https://www1.nyc.gov/site/doh/about/press/pr2017/pr078-17.page

¶
https://epi.publichealth.nc.gov/cd/hepatitis/HepatitisAandHIV.pdf

**
https://nychealth.tumblr.com/post/165278208281/health-alert-hepatitis-a-hepatitis-a-is-on-the
